# Histological and Histomorphometric Evaluation of Post-Extractive Sites Filled with a New Bone Substitute with or without Autologous Plate Concentrates: One-Year Randomized Controlled Trial

**DOI:** 10.3390/ma15010254

**Published:** 2021-12-29

**Authors:** Marco Tallarico, Erta Xhanari, Aurea Maria Immacolata Lumbau, Adela Alushi, Irene Ieria, Luca Fiorillo, Fausto Famà, Agron Meto, Edoardo Baldoni, Silvio Mario Meloni, Marco Cicciù

**Affiliations:** 1 School of Dentistry, University of Sassari, 07021 Sassari, Italy; me@studiomarcotallarico.it (M.T.); alumbau@uniss.it (A.M.I.L.); baldoni@uniss.it (E.B.); melonisilviomario@yahoo.it (S.M.M.); 2 Department of Implantology, Faculty of Dentistry, University of Aldent, 1031 Tirana, Albania; ertaxhanari@hotmail.com (E.X.); adela.alushi@ual.edu.al (A.A.); agron.meto@ual.edu.al (A.M.); 3 Private Practice, 00151 Rome, Italy; irene.ieria@gmail.com; 4 Department of Biomedical, Dental Science and Morphological and Functional Images, University of Messina, 98122 Messina, Italy; mcicciu@unime.it; 5 Multidisciplinary Department of Medical-Surgical and Odontostomatological Specialties, University of Campania “Luigi Vanvitelli”, 81100 Naples, Italy; 6 Department of Human Pathology in Adulthood and Childhood “G. Barresi”, University Hospital “G. Martino” of Messina, Via Consolare Valeria 1, 98125 Messina, Italy; ffama@unime.it

**Keywords:** socket preservation, L-PRF, grow factors, dental implants, biomaterials

## Abstract

The purpose of the present study was to evaluate the histological and histomorphometric characteristics of post-extraction sites grafted with decellularized bovine compact bone from bovine femur, mixed and unmixed with leukocyte- and platelet-rich fibrin after four months of healing. This study was designed as a randomized controlled trial of parallel groups. Patients in need of a single, implant-supported restoration to replace a hopeless tooth were recruited for tooth extraction and implant placement four months after socket preservation procedure. After tooth extraction, patients were randomly allocated to receive decellularized bovine compact bone from bovine femur, mixed and unmixed with leukocyte- and platelet-rich fibrin. After four months of healing, tapered implants were inserted with an insertion torque between 35 and 45 Ncm. Two months later, implants were loaded with screw-retained definitive crowns. Outcome measures were implant (ISR) and prosthesis (PSR) survival rates, complications, histological and histomorphometric analyses, radiographic marginal bone-level changes, and patients’ satisfaction. Clinical data were collected up to one year after tooth extraction and socket preservation procedures. Thirty patients were consecutively enrolled in the trial (15 in each group). Unfortunately, due to the COVID-19 pandemic, bone samples were collected only in 19 patients. Two implants failed before definitive prosthesis delivery (ISR 93.3%). No prosthesis failed (PSR 100%). Three complications were experienced in the control group. The mean bone percentage was 40.64 ± 18.76 in the test group and 33.40 ± 22.38 in the control group. The difference was not statistically significant (*p* = 0.4846). The mean soft tissue percentage was 32.55 ± 19.45 in the test group and 55.23 ± 17.64 in the control group. The difference was statistically significant (*p* = 0.0235). The mean residual graft was 24.59 ± 18.39 in the test group and 11.37 ± 12.12 in the control group. The difference was not statistically significant (*p* = 0.0992). Mean marginal bone loss, as well as patient satisfaction, showed no differences between groups. With the limitations of the present study, socket preservation with L-PRF mixed with decellularized bovine compact bone demonstrated favorable results, comparing with decellularized bovine compact bone from bovine femur alone. Further studies with larger sample size and longer follow-up are needed to confirm these preliminary results.

## 1. Introduction

Bone resorption subsequent to the extraction of a tooth and undisturbed wound healing may lead to loss of ridge volume and change in ridge shape, which may affect the prognosis of the implant therapy [1,2]. It has been shown that vertical bone resorption after tooth extraction varies from 11 to 22%, while the horizontal bone resorption varies from 29 to 63% within six months post-extraction [3]. In order to keep adequate bone and soft tissue levels to satisfy increasing demand for high esthetics and successful long-term results, several hard and soft tissue reconstruction techniques have been developed [4,5,6]. However, high costs and increased morbidity may be associated with these techniques. With the aim of reducing the need for guided bone regeneration after tooth extraction, socket preservation techniques have been developed. These techniques aim to minimize the shrinkage of hard and soft tissues during healing by grafting a bone substitute into the residual alveolar ridge, and a membrane to seal the socket [7,8]. Several grafting biomaterials, socket sealers, and growth factors have been used satisfactorily. However, there is currently no consensus on the gold standard technique and materials [9,10,11]. Resorbable and not resorbable materials were used to promote the bone healing and to compensate the physiological bone remodeling, due to the resorption of the bundle bone. Connective tissue graft, resorbable collagen-based matrices [2] and intentionally exposed not resorbable d-PTFE matrices were all used to seal the socket [11,12,13], reducing the microbiological contamination from the oral cavity [14].

Leukocyte- and platelet-rich fibrin (L-PRF) is centrifuged patient blood, which includes leukocytes in a high-density fibrin network, that can be used for several applications. The objective of this process is to gather and concentrate elements that may be used for therapeutic applications, including fibrinogen/fibrin, platelets, growth factors, leukocytes, and other forms of circulating cells [15]. Commonly, L-PRF is used to stimulate, improve and accelerate the natural process of healing on a surgical or wounded site, including the residual alveolar socket [16]. There is strong evidence from previous systematic reviews that the local application of PRF after a tooth extraction is a suitable method for reducing pain, swelling, and the incidence of alveolar osteitis [17,18]. However, positive effects of PRF in dental sockets on promoting bone regeneration are still controversial [17,18].

The purpose of this randomized controlled trial was to evaluate the histological and histomorphometric characteristics of post-extraction sites grafted with decellularized bovine compact bone from bovine femur (RE-BONE^®^ 0.5 g–0.25/1.0 mm granules, UBGEN Padova, Italy), mixed and unmixed with leukocyte- and platelet-rich fibrin after four months of healing. Moreover, the aim was to evaluate the clinical outcomes of implants placed after socket preservation techniques at the six months after loading follow-up (one year after tooth extraction). The null hypothesis that there are no differences in clinical, histological, and radiographic outcomes between groups was tested against the alternative hypothesis of differences. The manuscript was written according to the CONSORT guidelines.

## 2. Materials and Methods

### 2.1. Study Design

This research was designed as a randomized controlled multicenter trial conducted at the Aldent University, Tirana (Albania) and in a private clinic in Rome (Italy) between October 2018 and February 2021. Two expert surgeons (M.T. and E.X.), one in each center, performed all the surgical and prosthetic procedures. Participants were enrolled and treated in consecutive order as a part of routine treatments once their written consent had been obtained. Patients were informed before entering the study about the clinical procedures, materials used, potential risks, complications, and follow-up assessments required by the clinical trial. This study was carried out following the principles of the 2013 Declaration of Helsinki on experiments performed on human subjects. The ethics committee of the Aldent University (Tirana, Albania) approved the study protocol (protocol number 2/2018).

### 2.2. Inclusion/Exclusion Criteria

Any patient who required the extraction of at least one hopeless tooth between premolars, who was at least 18 years old and, therefore, able to sign the informed consent, and for whom an implant-supported prosthesis was indicated was considered eligible for this study. Hopeless teeth were judged as follows: furcation involvement > II; mobility > II; PPD > 6 mm, with percentage of alveolar bone loss/root length ≥70%; persistent radiographic pathology and/or symptoms (e.g., pain, fistula, chronic infection) of endodontic origin and an uncertain prognosis; restorability [19,20,21].

No patient was admitted to the study if at least one of the following exclusion criteria was present: general contraindications to implant surgery; compromised sockets (buccal dehiscence ≥ 3 mm (Class II or III by Elian et al. [22]) and/or abscess; pregnancy or breastfeeding; untreated periodontitis (plaque index (PI) and bleeding on probing (BoP) ≥ 25%); severe bruxism or clenching; immunosuppression; previous history of head–neck irradiation; uncontrolled diabetes; severe smokers (>10 cigarettes/day); poor oral hygiene and motivation; present or past intravenous bisphosphonate treatment; substance abuse (alcohol, drugs); and psychiatric disorders. On the basis of the clinical, radiographic, and anamnestic data collected, it was decided whether or not to include the patients in the study according to the criteria described above.

### 2.3. Surgical and Prosthetic Protocols

Patients included in the study underwent initial periodontal therapy, including oral hygiene instructions. Then, all the included patients underwent periapical radiograph prior to surgery. Antibiotic prophylaxis (amoxicillin 2 g) was prescribed one hour before starting surgery and all patients were treated under local anesthesia with articaine hydrochloride with epinephrine 1:100,000 (Orabloc, Pierrel, Milan, Italy). Hopeless teeth were extracted with a minimally invasive technique, without a flap. After tooth extraction, the integrity of the socket was checked using a periodontal probe. Patients were assigned to the test or control group according to the predefined randomization table by opening an opaque envelope containing the randomization code. In both groups, the residual socket was cleaned using an ultrasound insert. After that, in the test group, a blood sample was collected in a single 9 mL plastic tubes for each patient, and L-PRF was prepared according to the manufacturer’s instruction at 2700 rpm for 12 min (IntraSpinTM, Intra-Lock International-Inc., Boca Raton, FL, USA). After that, the fibrin clot was pressed (Xpression^TM^ Components, Intra-Lock International-Inc., Birmingham, AL, SUA) until it produced a thick, round fibrin matrix that was gently cut into small pieces in a sterile dish. The prepared autologous L-PRF was mixed with decellularized bovine compact bone from bovine femur (RE-BONE^®^ 0.5 g–0.25/1.0 mm granules, UBGEN, Padova, Italy) and grafted into the alveolus, up to the soft tissue margin. In the control group, only decellularized bovine compact bone (BM RE-BONE 01B; 0.5 g–0.25–1 mm granules, UBGEN, Padova, Italy) was used. In both groups, heterologous type 1 collagen (Condress, Smith and Nephew Srl, Agrate Brianza, Italy) was used to seal the socket and cover the graft. Collagen was stabilized with 4-0 adsorbable sutures (Vicryl, Ethicon Johnson and Johnson International, Sint-Stevens-Woluwe, Belgium). Antibiotic (1 gr of Amoxicillin) was continued every 12 h for five days. Patients were instructed on taking medications and limiting oral hygiene techniques at the surgical site, using only soft brushes. Regular brushing techniques were instead performed in the remaining areas of the mouth and rinsing with 0.2% chlorhexidine twice a day for two weeks was recommended. Sutures were removed 10–14 days after surgery.

**Figure 1:**
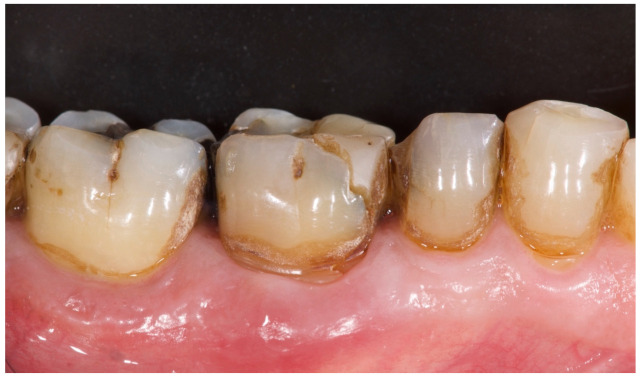
Preclinical situation of patient number 3. Lower right first molar needed to be extracted due to a vertical fracture.

**Figure 2:**
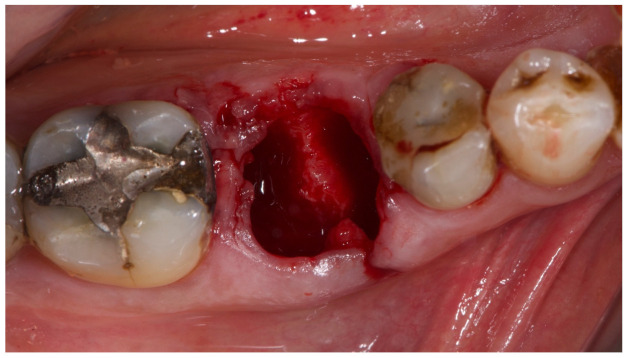
Atraumatic tooth extraction.

**Figure 3:**
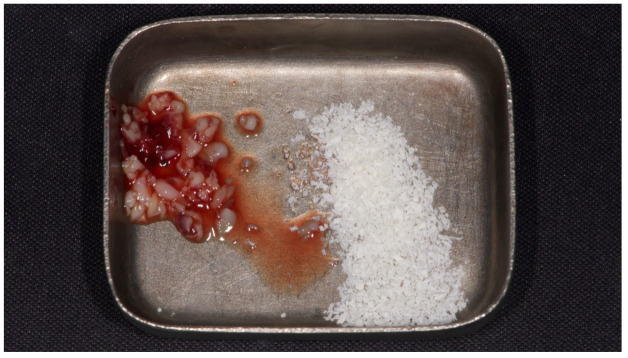
L-PRF (left) and decellularized bovine compact bone from bovine femur (right).

**Figure 4:**
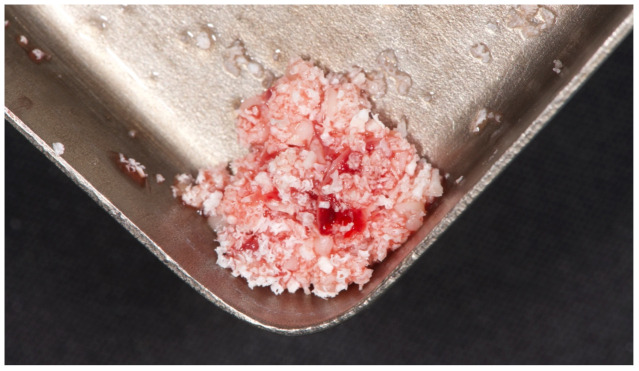
L-PRF mixed with decellularized bovine compact bone from bovine femur.

**Figure 5:**
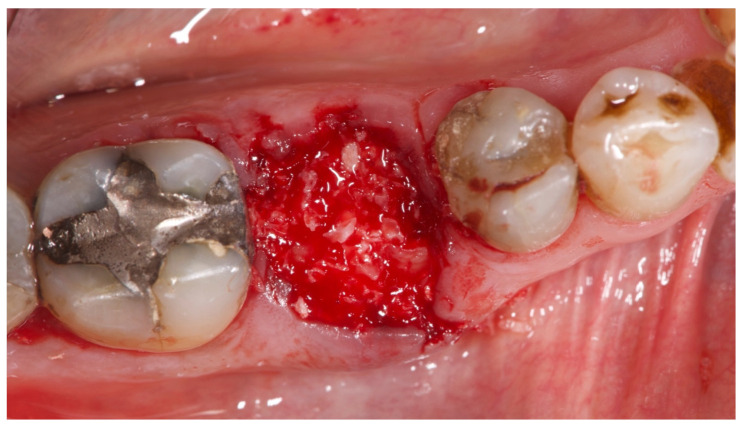
Residual socket grafted with the mixture of L-PRF and bone substitute.

**Figure 6:**
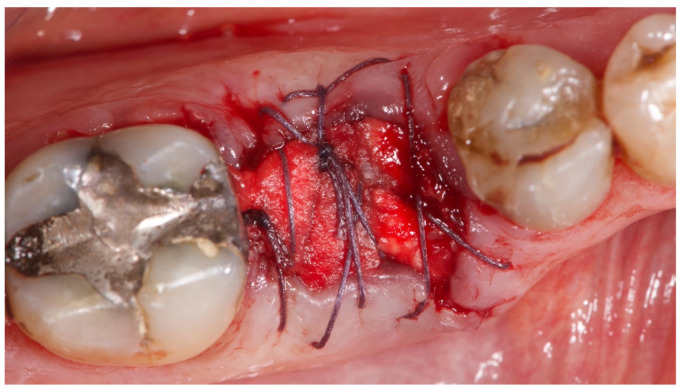
Residual socket sealed with collagen type one and resorbable suture.

**Figure 7:**
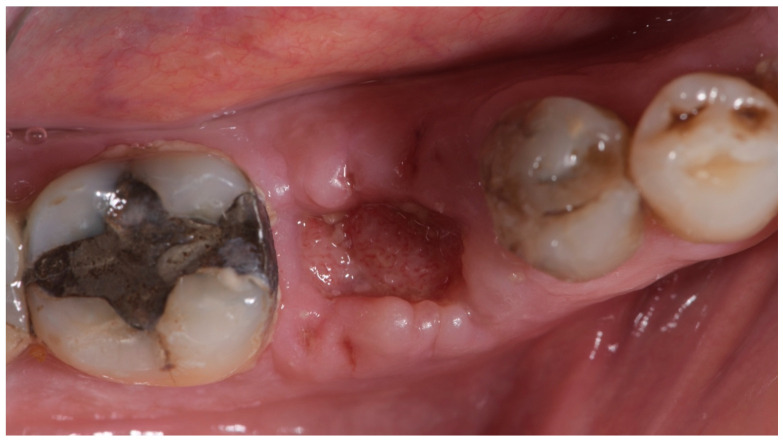
Occlusal view 2 weeks after tooth extraction and socket preservation procedure.

**Figure 8:**
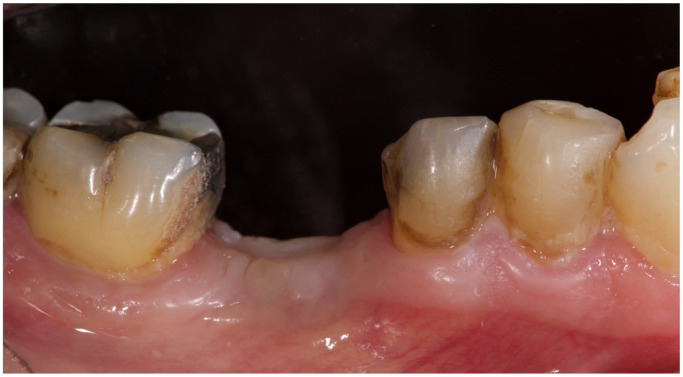
Lateral view four months after tooth extraction and socket preservation procedure.

**Figure 9:**
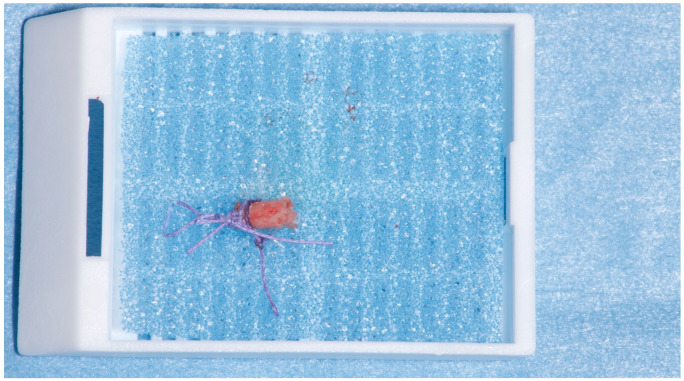
Example of collected sample. A suture was placed to immobilize the sample and to define the most occlusal part of the sample itself.

Four months after tooth extraction and socket preservation, all the patients underwent a cone beam computer tomography scan (CBCT, Cranex 3Dx, Soredex, KaVo Kerr Group, Sesto San Giovanni, Milano, Italy) immediately before implant placement. This allowed the quantitative evaluation of the bone volume maintained after socket preservation procedure. After residual bone volume was judged adequate, the patients received the planned dental implants. Implant surgery was performed under local anesthesia, following a full thickness flap elevation. Before implant site preparation, a trephine bur drill of 3.0 mm diameter (external diameter) was used to collect the bone samples. Tapered, sandblasted/acid-etched, bone level implants, featured with 11° conical connection (Osstem TSIII, Osstem Implant Ltd., Seoul, Korea), were placed according to the bone density and the manufacturer’s instructions. Implants were placed according to a one-stage protocol and early loaded two months after their placement. All the implants received a single, screw-retained, zirconia ceramic crown. An explanatory case is reported in Figure 1, Figure 2, Figure 3, Figure 4, Figure 5, Figure 6, Figure 7, Figure 8 and Figure 9.

### 2.4. Clinical Outcome Assessments

- Implant (ISR) and prosthetic (PSR) survival rates, and any biological and technical complications were assessed and treated by the same surgeons, including any conditions that do not allow for conventional implant placement.
- Histological and histomorphometric evaluations of the post-extraction sites comparing the two groups (test and control) were carried out. Samples were collected and fixed in formaldehyde 4% prefilled containers. After that, samples were labeled and processed by a blinded pathologist, using a pre-published, standardized protocol [9].
- Marginal bone loss (MBL) was defined as the difference between marginal bone levels (the distance between the most coronal part of the implant and the first bone-to-implant contact), evaluated on standardized periapical radiographs taken at implant placement and 6 months after definitive prosthesis delivery by a blinded assessor. The mean value between the mesial and distal marginal bone levels was used in the statistical analysis.
- The patient’s degree of satisfaction during this was assessed through a short survey, administered six months after definitive prosthesis delivery by a blinded independent assessor not previously involved in the study. Possible answers were “yes” or “no”.
- Are you satisfied with the function of your implant-supported prosthesis?
- Are you satisfied with the aesthetic outcome of your implant-supported prosthesis?
- Would you undergo the same therapy again?

### 2.5. Randomization and Statistical Analysis

A prior sample size was not performed. It was decided to enroll 15 patients at each center, based on the possible contribution. The randomization list was generated using a dedicated, online software (https://www.random.org/lists/ (accessed on 9 June 2018)). Randomized sites were coded and placed in a closed opaque envelope. Operators were aware of the patient’s allocation (test or control group) only after opening the envelope, immediately after tooth extraction.

Statistical analyses were carried out in order to identify any differences between groups. A statistician with experience in dentistry performed all the analyses without knowing the group codes. Mean values and standard deviations were calculated for each continuous outcome (histomorphometric and radiographic parameters) using Numbers for Mac (version 11.0 (7030.0.94), Apple Inc., Cupertino, CA, USA). Comparison between groups was carried out using the Independent Samples *t*-test (Numbers for Mac version 11.0 (7030.0.94)). Implant and prosthetic survival rates, complications, and patient’s degree of satisfaction (dichotomous outcomes) were compared using the Fisher’s exact test. All statistical comparisons will be conducted at the 0.05 significance level.

## 3. Results

**Figure 10:**
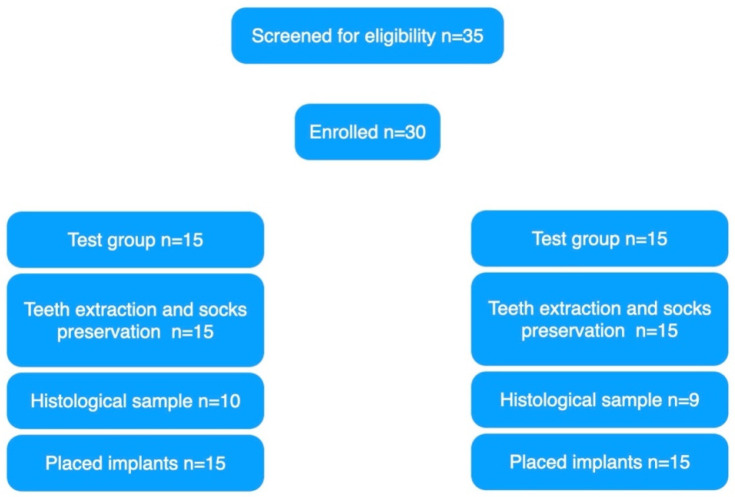
Participant flow diagram according to the CONSORT guidelines.

Thirty-five patients were screened for eligibility, but four patients (two at each center) could not be enrolled in the trial because they refused to have bone sample. One more patient from the Albanian center refused to sign the research informed consent. Finally, 30 patients were considered eligible and were consecutively enrolled in the trial (15 in each group). All patients were treated according to the allocated interventions, and no patient dropped out. Data of all patients were evaluated in the statistical analyses. Nevertheless, due to the COVID-19 pandemic, the main deviations from the original research protocol were that the bone samples were not collected in 11 patients (four at the Italian center and seven at the Albanian center) according to the prevention measures aimed to reduce the risk of virus transmission. Finally, 30 patients were treated but only 19 histological samples were collected and analyzed. There is no unbalancing between groups (Table 1). A graph of the enrolled patients is reported in Figure 10.

Two implants failed before definitive prosthesis delivery, one for each group (ISR 93.3%). The implants were replaced after an undisturbed healing period of three months. No further implant failure was experienced. No prosthesis failed (PSR 100%). Three complications were experienced in the control group. In two cases, a supporting guided bone regeneration was needed at the time of implant placement due to thin buccal bone. In the third case, implant was placed 2 mm below the bone crest for the same reason. Nevertheless, the difference between groups was not statistically significant (*p* = 0.2241).

**Figure 11:**
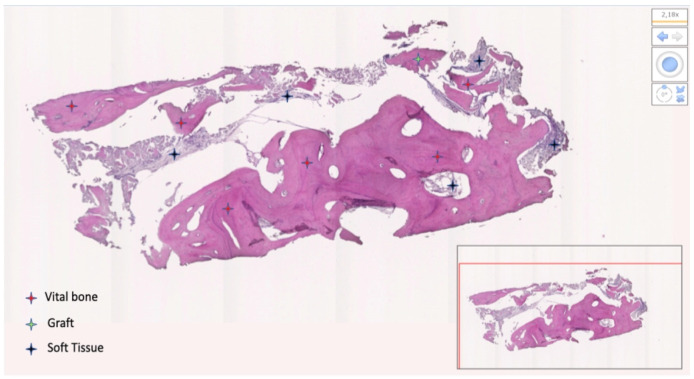
Bone sample taken at the lower right first molar. Patient (number 3) received L-PRF mixed with decellularized bovine compact bone from bovine femur (test group). The bone was 76.65%; soft tissue was 8.73%; residual graft was 15.62%.

**Figure 12:**
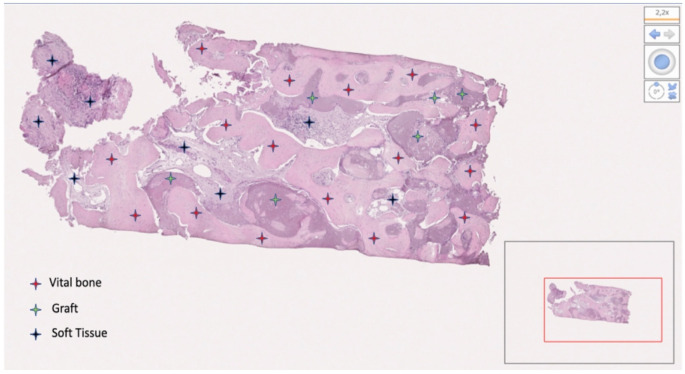
Bone sample taken at the upper right first premolar. The patient (number 13) received decellularized bovine compact bone from bovine femur alone. The bone was 46.19%; soft tissue was 34.61%; residual graft was 19.20%.

**Figure 13:**
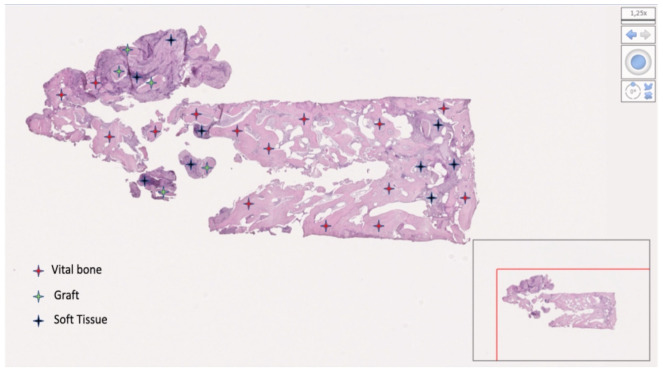
Bone sample taken at the upper right second premolar. The patient (number 10) received L-PRF mixed with decellularized bovine compact bone from bovine femur. The bone was 48.22%; soft tissue was 48.92%; residual graft was 2.86%.

**Figure 14:**
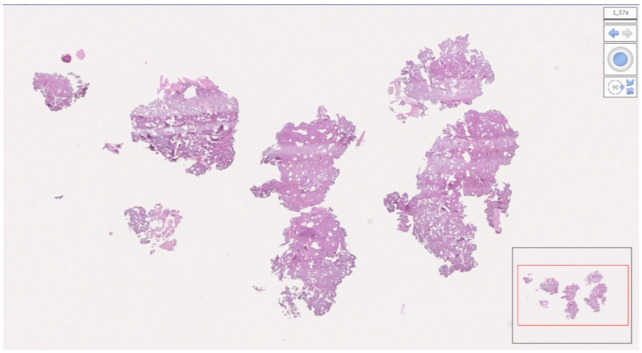
Bone sample taken at the upper right second molar. The patient (number 16) received L-PRF mixed with decellularized bovine compact bone from bovine femur. The sample was not assessable.

From the 19 collected samples, two were not processable (one in each group). Finally, nine samples in the test group and eight in the control group were analyzed. The mean bone percentage was 40.64 ± 18.76 (95% CI 32.20 to 49.08) in the test group (n = 9) and 33.40 ± 22.38 (95% CI 23.33 to 43.46) in the control group (n = 8). The difference was not statistically significant (*p* = 0.4846). The mean soft tissue percentage was 32.55 ± 19.45 (95% CI 23.81 to 41.30) in the test group (n = 9) and 55.23 ± 17.64 (95% CI 47.30 to 63.17) in the control group (n = 8). The difference was statistically significant (*p* = 0.0235). The mean residual graft was 24.59 ± 18.39 (95% CI 16.32 to 32.85) in the test group (n = 9) and 11.37 ± 12.12 (95% CI 5.92 to 16.82) in the control group (n = 8). The difference was not statistically significant (*p* = 0.0992). Data are reported in Table 2 and Figure 11, Figure 12, Figure 13 and Figure 14.

All the implants were placed at the crestal bone level or slightly below. Six months after definitive prosthesis delivery, there is not any significance difference between groups. Data are reported in the Table 3.

The patient’s degree of satisfaction is reported in Table 4.

## 4. Discussion

The aim of this study was to compare clinical, histological, and radiographic outcomes of post-extraction sockets grafted with decellularized bovine compact bone from bovine femur, mixed or unmixed with leukocyte- and platelet-rich fibrin, one year after tooth extraction. The use of PRP in dentistry dates to the 1990s; this component is present in two different formulations: PRP or PRF. The null hypothesis that there are no differences in clinical, histological, and radiographic outcomes between groups was partially rejected. Although there is no significant difference in clinical and radiographic outcomes, the histomorphometric analyses showed an increased amount of bone tissue and significantly less soft tissue in the sites grafted with decellularized bovine compact bone mixed with L-PRF. These results are in agreement with previous animal and human studies that showed an increase in new bone formation and a positive effect on early bone healing using platelet-rich fibrin preparation in combination with bone substitute [23,24,25]. Nevertheless, the exact mechanism and results of combining PRF with bone grafts on the bone healing process is still unknown. Even if some preliminary results could be drawn, the main limitation of this study was the small sample size, due to a prior sample size not being calculated. Moreover, the COVID-19 pandemic underpowered the initial sample size. Other limitations could be the short follow-up. Nevertheless, the main purpose of this study was to evaluate histological and histomorphometric features of socket healing grafted with decellularized bovine compact bone from bovine femur, with and without L-PRF.

The use of biomaterials is predictable in oral and maxillofacial surgery. This is thanks to the continuous improvements and the continuous formulation of biomaterials. The gold standard, repeatedly defined by some studies in the literature, does not actually exist. This would be a material that has multiple properties: osteoconductive, osteoinductive, osteoproliferative. Nowadays, prosthetically driven implant placement represents the gold standard in implant dentistry. Ideally, up to 2 mm of bone should be present 360° around dental implants. However, most of the alveolar bone volume could be lost after tooth extraction [3]. A commonly applied socket preservation technique involves a flapless approach and bone grafting of the residual socket to prevent bone loss immediately after extraction [7,8]. However, it was demonstrated that a lack of socket sealing could lead to clinical failures [26,27]. To overcome these limitations, various soft tissue management techniques have been proposed, including advanced coronal flap [28], epithelial-connective soft tissue graft [29], resorbable porcine collagen matrix [11], or intentionally exposed, not resorbable membranes [12]. Autogenous materials are still considered the gold standard procedure with proven clinical success; nevertheless, high morbidity of the donor site with patient discomfort has been reported [30,31]. On the other hand, xenografts are expensive, and its contribution is still controversial [11].

Leukocyte- and platelet-rich fibrin (L-PRF) are activated gel preparation that could be used as efficient adjuvants for tissue soft repair, modulating various steps of the soft tissue healing process, such as hemostasis and neoangiogenesis [32]. Growth factors demonstrated positive effects, even when mixed with biomaterials. Calcium phosphate cements, if mixed with BMP-2, PDGF, FGF, could also help solve problems in cases of complex bone regeneration, such as those of peri-implant defects. It is proved that FD-PRF improves the expression of transcription factors compared to PRF if it is used for alveolar bone regeneration in animals. FD-PRF presents an enormous advantage, it could be stored at room temperature for several months and reconstituted on demand. PRP could have an inhibitory function against bacteria growth; platelets express receptors of the toll-like receptor family (TLR), which bind bacterial targets and favor microbicidal proteins release. FD-PRP is promising to functionalize bio-printed tissues. It is important to state grafting guidelines for this product regarding the titration of antibodies. In a recent randomized controlled trial with a splits-mouth design, Sammartino and colleagues demonstrated that the use of L-PRF in post-extraction sockets was able to reduce the postoperative pain and to promote the soft tissue healing process. The latter can potentially reduce the early adverse effects of the inflammation process [33,34,35]. Looking in this direction, mixing the bone substitute with the same part of L-PRF may be a viable option to prevent complications, pain, and also to reduce the amount of soft tissue before implant placement. In the present study, a low-cost, heterologous type 1 collagen was used to seal the socket. It is the authors’ opinion that type 1 collagen could be sufficient to stabilize the coagulum during the first weeks of healing, particularly if L-PRF is used as adjuvant material. However, another limitation of the present study is that the amount of keratinized tissue was not calculated.

In the present study, all the implants were placed as planned. Only two implants failed at the second-stage surgery, due to loss of osseointegration. Nevertheless, three complications were reported in the control group. In all of these three cases, conventional implant placement was not possible. Two cases required guided bone reconstruction at the same time of implant placement. In the last case, the implant was placed deeper to avoid bone management. In all of these cases, L-PRF was not used as adjunctive material during the socket preservation technique. However, in order to understand possible reasons, further studies evaluating tridimensional bone volume changes, as well as thickness of the soft tissues, are needed [34].

## 5. Conclusions

With the limitations of the present randomized controlled trials, due to sample size, socket preservation with L-PRF mixed with decellularized bovine compact bone could represent favorable results comparing with decellularized bovine compact bone from bovine femur alone. Further studies with larger sample size and longer follow-up are needed to confirm these preliminary results and to develop predictable surgical techniques and biomaterials.

## Figures and Tables

**Table 1:** Patient and implant characteristics between groups.

	Group 1 (n = 15)	Group 2 (n = 15)	*p*-Value
Age	52.1 ± 11.5	49.2 ± 12.3	0.515
Sex Male/Female	5/10	5/10	1.0
Smoking	2	3	1.0
Histological samples	10	9	1.0
Mean implant diameter	4.3 ± 0.5	4.3 ± 0.5	1.0
Mean implant length	9.7 ± 1.6	10.1 ± 1.7	0.521
Maxilla/Mandible implants	10/5	12/3	0.682
Implant failure	1	1	1.0
Prosthetic failure	0	0	1.0
Complications	0	3	0.2241

**Table 2:** Histomorphometric analysis between groups.

	Group 1 (n = 9)	Group 2 (n = 7)	*p*-Value
Bone %	40.64 ± 18.76	33.40 ± 22.38	0.4846
Soft tissue %	32.55 ± 19.45	55.23 ± 17.64	0.0235
Graft %	24.59 ± 18.39	11.37 ± 12.12	0.0992

**Table 3:** Marginal bone levels between groups.

	Group 1 (n = 15)	Group 2 (n = 15)	Difference (MBL)
Implant placement	0.02 ± 0.04	0.30 ± 0.09	0.28 ± 0.09
Six months after prosthesis delivery	0.01 ± 0.02	0.31 ± 0.11	0.30 ± 0.12
*p*-Value	0.5573	0.7902	0.6636

**Table 4:** Patient’s degree of satisfaction (yes/no).

	Group 1 (n = 15)	Group 2 (n = 15)	*p*-Value
Are you satisfied with the function of your implant-supported prosthesis?	15/0	15/0	1.0
Are you satisfied with the aesthetic outcome of your implant supported prosthesis?	13/2	14/1	1.0
Would you undergo the same therapy again?	15/0	15/0	1.0

## Data Availability

Raw data and all the histologies are available by a written request addressed to the corresponding author (M.T.) or the company (Ubgen S.r.l.).
